# Nuclear detection of Y-*box *protein-1 (YB-1) closely associates with progesterone receptor negativity and is a strong adverse survival factor in human breast cancer

**DOI:** 10.1186/1471-2407-9-410

**Published:** 2009-11-24

**Authors:** Edgar Dahl, Abdelaziz En-Nia, Frank Wiesmann, Renate Krings, Sonja Djudjaj, Elisabeth Breuer, Thomas Fuchs, Peter J Wild, Arndt Hartmann, Sandra E Dunn, Peter R Mertens

**Affiliations:** 1 Molecular Oncology Group, Institute of Pathology, Medical Faculty, RWTH Aachen University, Aachen, Germany; 2 Department of Nephrology and Hypertension, Otto-von-Guericke-University Magdeburg, Magdeburg, Germany; 3 Department of Nephrology and Immunology, Medical Faculty, RWTH Aachen University, Aachen, Germany; 4 Department of Computer Science, ETH Zurich, Zurich, Switzerland; 5 Institute of Surgical Pathology, University Hospital Zurich, Zurich, Switzerland; 6 Department of Pathology, University of Erlangen, Germany; 7 Laboratory for Oncogenomic Research, Department of Pediatrics, Child and Family Research Institute, University of British Columbia, Vancouver, British Columbia, Canada

## Abstract

**Background:**

Y-box binding protein-1 (YB-1) is the prototypic member of the cold shock protein family that fulfills numerous cellular functions. In the nucleus YB-1 protein orchestrates transcription of proliferation-related genes, whereas in the cytoplasm it associates with mRNA and directs translation. In human tumor entities, such as breast, lung and prostate cancer, cellular YB-1 expression indicates poor clinical outcome, suggesting that YB-1 is an attractive marker to predict patients' prognosis and, potentially, is suitable to individualize treatment protocols. Given these predictive qualities of YB-1 detection we sought to establish a highly specific monoclonal antibody (Mab) for diagnostic testing and its characterization towards outcome prediction (relapse-free and overall survival).

**Methods:**

Hybridoma cell generation was carried out with recombinant YB-1 protein as immunogen and Mab characterization was performed using immunoblotting and ELISA with recombinant and tagged YB-1 proteins, as well as immunohistochemistry of healthy and breast cancer specimens. Breast tumor tissue array staining results were analyzed for correlations with receptor expression and outcome parameters.

**Results:**

YB-1-specific Mab F-E2G5 associates with conformational binding epitopes mapping to two domains within the N-terminal half of the protein and detects nuclear YB-1 protein by immunohistochemistry in paraffin-embedded breast cancer tissues. Prognostic evaluation of Mab F-E2G5 was performed by immunohistochemistry of a human breast cancer tissue microarray comprising 179 invasive breast cancers, 8 ductal carcinoma *in situ *and 37 normal breast tissue samples. Nuclear YB-1 detection in human breast cancer cells was associated with poor overall survival (p = 0.0046). We observed a close correlation between nuclear YB-1 detection and absence of progesterone receptor expression (p = 0.002), indicating that nuclear YB-1 detection marks a specific subgroup of breast cancer. Likely due to limitation of sample size Cox regression models failed to demonstrate significance for nuclear YB-1 detection as independent prognostic marker.

**Conclusion:**

Monoclonal YB-1 antibody F-E2G5 should be of great value for prospective studies to validate YB-1 as a novel biomarker suitable to optimize breast cancer treatment.

## Background

Since the seminal description of Janz et al. [1] that high YB-1 expression levels in breast cancer and surrounding tissues are indicative for poor outcome, follow-up studies have extended these findings to larger cohorts and other cancer entities, including non-small cell lung cancer [2], ovarian cancer [3], prostate cancer [4], and synovial sarcoma [5]. A recent study confirmed the unfavorable outcome of patients with YB-1 expression in breast cancer tissue with a large cohort of 4049 cases over an observation period of 20 years, which reached statistical significance in nearly all subgroups [6]. Furthermore, Bargou et al. [7] reported that nuclear localization of YB-1 was associated with P-glycoprotein expression in human primary breast cancers, other studies have shown a co-expression of YB-1 and P-glycoprotein in osteo- and synovial sarcoma as well as breast, ovarian and prostate cancer [3,5,8-11].

Cold-shock proteins like the Y-box binding (YB) protein fulfill pleiotropic functions, e.g. regulation of target genes, (pre-)mRNA splicing and translation [12-16]. YB-1 is a multifunctional protein with fascinating roles in cell biology. First and utmost, YB-1 serves multiple roles in gene transcription with numerous target genes involved in DNA replication and proliferation [17-22]. Overexpression of YB-1 via a transgene in a mouse model induced the development of breast cancers of many histological types [23], suggesting that YB-1 is oncogenic. Reports confirm the relevance of YB-1 expression for EGF-independent breast cancer cell growth [24]. Following its original cloning as binding activity of the EGF receptor promoter [25], Dunn et al. recently reported that YB-1 is a strong *trans*-stimulator of EGF receptor expression in breast cancer cells [26].

A predominant nuclear localization of YB-1 protein goes along with intrinsic expression of putative oncogenes [1,4,24,27], response to oxidative stress and coordination of DNA excision repair [28]. On the other hand, there is evidence for a prominent role of YB-1 as RNA-binding molecule and constituent of cytoplasmic major messenger ribonucleoprotein particles (in this context denoted p50) [29,30]. Studies addressing the question what regulates YB-1 expression, its subcellular localization and potential protein modifications have shed some light on underlying mechanisms. The transcription of YB-1 is upregulated by basic helix-loop-helix transcription factor Twist [31]. Twist itself is an immediate target of *signal tranducers and activator of transcription *(STAT)-3, which may be activated by epidermal growth factor receptor signaling [32]. *Knock-down *of YB-1 completely abrogated the proliferative effect of Twist, emphasizing the fundamental role that YB-1 plays for the EGF receptor axis. Furthermore, serine 102 of YB-1 protein is a direct target of protein kinases B (AKT) and RSK [33], both of which are signaling cascades involved in cell transformation, proliferation and anchorage-independent growth [17,27,34].

A general evaluation of YB-1 expression in breast cancer patients is limited due to lack of a suitable antibody of unrestricted quantity and defined quality, preferably monoclonal. This could potentially allow to diversify their relative risk profile and chemotherapy sensitivity [7,35]. Furthermore, most polyclonal antibodies, like the ones used for breast cancer tissue by Janz et al. [1] and Habibi et al. [6], predominantly detect YB-1 in the cytoplasm, whereas YB-1 protein activities relating to chromosomal instability and gene regulation must take place within the nuclear compartment [36].

In the following we present data on a newly established monoclonal YB-1 antibody that is characterized as suitable for immunohistochemistry in paraffin-embedded tissue. The expression of YB-1 by means of this antibody was analyzed by immunohistochemistry in a cohort of breast cancer specimens. For all tumour specimens analyzed, full histopathological and clinical follow-up data were available, allowing uni- and multivariate analyses of nuclear YB-1 expression in correlation to well-established factors of breast cancer prognosis (grade, nodal status, HER2, estrogen receptor (ER) status, progesterone receptor (PR) status). Thereby we characterized the Mab as a novel tool that can be used in breast cancer prognosis and therapy stratification.

## Methods

### Mab generation

Mouse work was in full compliance with the guidelines for animal care and was approved by the animal care committee from the government. Female BALB/c mice were immunized by intraperitoneal and intravenous injection of 100 μg recombinant YB-1 full-length protein emulsified in equal volume of Freund's adjuvant (Gibco-BRL life technologies, Karlsruhe, Germany) followed by a booster injection six weeks later containing 10 μg of antigen in Freund's adjuvant. The response was assessed by dot blot assay with recombinant YB-1 protein. Three days after the last booster injection, spleen cells were obtained and fused with X63-Ag/653 (BALB/c) mouse myeloma cells and propagated according to standard procedures. The fused cells were resuspended in DMEM medium (Gibco BRL life technologies) supplemented with 10% bovine calf serum, 100 U/ml penicillin, 100 μg/ml streptomycin, 50 μM hypoxanthine, 160 μM thymidine, and 400 nM aminopterin. Aliquots of the cell suspension (100 μl) were dispensed into 96-well plates and incubated at 37°C/5% CO_2_. Every week the medium was replaced with fresh selection medium supplemented with recombinant interleukin-6 (100 U/ml). After two weeks the medium was replaced by HT-medium (DMEM medium (Gibco BRL life technologies) supplemented with 10% bovine calf serum, 100 U/ml penicillin, 100 μg/ml streptomycin, 100 μM hypoxanthine and 160 μM thymidine and another week later cells were cultured in standard DMEM medium (4500 mg/l D-Glucose supplemented with GlutaMax (Gibco BRL life technologies) with 10% bovine calf serum, 100 U/ml penicillin and 100 μg/ml streptomycin. Hybridoma supernatants from 96-well plates were screened by dot blot assay with recombinant YB-1 protein for specific antibody synthesis. The positive-tested hybridoma cells were single-cell cloned twice by limited dilution in standard medium as described [37]. Immunoglobulins in the hybridoma cell culture supernatants were purified using protein A sepharose or protein G sepharose columns according to the immunoglobulin isotype (ÄKTA_FPLC _systems, Amersham Biosciences, Freiburg, Germany). F-E2G5 was of IgG_2b _isotype. Purified monoclonal antibodies were conjugated to biotin as described [38].

### Plasmids, cell line and transfection

Plasmids encoding for GFP and YB-1-GFP fusion proteins (pcDNA6/V5-His-YB-1-GFP) have been described previously [20]. HEK293T cells were cultured in DME-medium containing 10% fetal bovine serum, 100 U/ml penicillin, 100 μg/ml streptomycin and 2 mM L-glutamine. Transient transfections of HEK293T cells with vectors were performed by means of calcium phosphate precipitation methodology using purified endotoxin-free plasmid DNA preparations. HEK293T cells expressing the respective proteins were harvested 48 h after transfection and cytoplasmic and nuclear fractions were prepared by cell lysis in buffer A (10 mM HEPES [pH 7.9], 1.5 mM MgCl_2_, 10 mM KCl, 0.2 mM PMSF, 1 mM sodium vanadate, 0.5 mM DTT) for 10 min on ice, followed by centrifugation at 2.000 rpm for 1 minute. The supernatant corresponds to soluble cytoplasmic proteins and contains GFP and YB-1-GFP proteins.

### Western Blotting

SDS-PAG electrophoresis was performed with protein extracts containing GFP or YB-1-GFP protein under reducing and non-reducing conditions. Proteins were separated on 12% SDS-PAG and transferred to nitrocellulose membranes (Schleicher-Schuell, Dassel, Germany). Membranes were blocked in TTBS (10 mM Tris-HCl [pH 8.0], 150 mM NaCl, 0.2% Tween-20) containing 5% non-fat dry milk for 1 h at room temperature. After three washing steps in TTBS, blots were incubated with biotin-labeled YB-1 Mab F-D2G5 diluted 1:2,000 and StreptABComplex diluted 1:50 (DAKO Cytomation, Glostrup, Denmark) was added. Following two more washes in TTBS the peroxidase reaction was visualized by ECL system (Amersham, Freiburg, Germany). Immunoprecipitation experiments were performed with the indicated antibodies that were incubated with pre-cleared pansorbin.

### ELISA

Recombinant YB-1 protein was expressed in *E. coli *and affinity purified as previously described [39]. For ELISA 96-well polystyrene plates were incubated with 200 μl of recombinant YB-1 protein solution (3 ng/μl) in coating buffer (15 mM Na_2_CO_3_, 35 mM NaHCO_3_) overnight at 4°C. After four washing steps with phosphate-buffered saline with 0.05% Tween-20 (50 mM sodium phosphate, 3 mM potassium phosphate, 150 mM sodium chloride [pH 7.4]), blocking with 2.5% low fat milk powder in PBS was performed at 37°C for 1 h. After four additional washing steps Mabs dissolved in washing buffer at 1:50 were added to each well (200 μl) and incubated another hour at 37°C and washed four times. Bound Mabs were detected either by incubation with 100 μl HRP-conjugated polyclonal anti-mouse IgG diluted 1:1,000 in PBS/Tween (non-biotin-labelled Mabs) or by incubation with 100 μl StrepABComplex diluted 1:50 (DAKO Cytomation, Glostrup, Denmark) with biotin-labelled Mabs for 1 h at 37°C and developed with ABTS (2,2'-azino-di-3-ethylbenzthiazoline-6-sulfonic acid)/H_2_O_2_. One row was used as blank containing immobilised YB-1 protein but with no addition of Mabs.

### Immunohistochemistry

Immunohistochemistry was performed as described recently [40] using an established breast tissue microarray [41]. The breast tissue samples of this tissue microarray were obtained from patients treated by primary surgery for breast cancer at the Department of Gynecology, University Hospital Regensburg, Germany, with institutional review board approval. All patients gave informed consent to the study for retention and analysis of their tissue for research purposes. Briefly, tissue sections were deparaffinized and rehydrated, and the endogenous peroxidase activity was quenched by treatment with 3% H_2_O_2_. Antigen retrieval was performed by pretreatment in citrate buffer [pH 6.0] in a microwave oven (three times for 8 minutes at 600 W). Slides were incubated with a 1:25 dilution of monoclonal biotin-labeled YB-1 antibody in 2% milk powder dissolved in PBS in a humidified chamber overnight at 4°C. After another wash Vectastain avidin-biotin complex (Vector Laboratories, California, USA) was added for 10 minutes. Immunostaining was visualized using 3,3'-diaminobenzidine tetrahydrochloride (DAB, Sigma, Deisenhofen, Germany) and hydrogen peroxide for 5 minutes. Finally, sections were counterstained with Mayer's hematoxylin. The primary antibody was omitted for negative controls. Tissue microarray slides were analyzed by a pathologist without knowledge of clinicopathological parameters of the tumors. Nuclear YB-1 staining was simply scored as positive (1) or negative (0), depending on visual inspection of tumor nuclei under high magnification (400×). The decision to score the staining pattern according to a dichotomous criterium, nuclear versus non-nuclear staining seemed justified as the pattern was evident throughout all specimens. There was no tissue with restricted focal nuclear staining. Cytoplasmic YB-1 staining was scored according to the immunoreactivity score (IRS) system developed by Remmele and Stegner [42], subsequently tumors were grouped into low expressers (IRS 0-3) and high expressers (IRS 4-12). For comparison of the F-E2G5 tissue staining pattern with peptide-derived affinity-purified YB-1 antibodies, the following antibodies were utilized: YB-1#1 has been generated by immunization and purification with a peptide corresponding to an N-terminal YB-1 epitope and was purchased from antibodies-online; YB-1#2 has been described by Janz et al. [1])

### Statistical analyses of tissue microarray data

Statistical analyses were performed with SPSS version 17.0 (SPSS, Chicago, IL). Differences were considered statistically significant when p < 0.05. Contingency table analyses and two-sided Fisher's exact tests were used to study the statistical association between clinicopathologic and immunohistochemical variables. Recurrence-free and disease-specific survival curves comparing patients with or without any of the factors were calculated using the Kaplan-Meier method, with significance evaluated by two-sided log-rank statistics. Disease-specific survival and recurrence-free survival were measured from time of surgery. For the analysis of recurrence, patients were censored at the time of their last tumor-free clinical follow-up appointment. For disease-specific survival analysis, patients were censored at the time of their last tumor-free clinical follow-up appointment or at their date of death not related to the tumor. A stepwise multivariable Cox regression model was adjusted, testing the independent prognostic relevance of nuclear YB-1 immunoreactivity. The limit for reverse selection procedures was p = 0.01. The proportionality assumption for all variables was assessed with log-negative-log survival distribution functions.

### Multiple logistic regression model

Regression analyses were conducted with R 2.8 (r-project.org). The binary response variable y is regressed on the explanatory variables, x_1_, x_2_, ..., x_q_. The model for n observations is defined by **ln(p/(1-p)) **= **Xβ **+ **ε**, where **ε **contains the residual error terms and p = Pr(y = 1). The parameters are estimated by maximum likelihood estimation. The regression coefficients β1, β_2_, ..., βq give the change in the response variable corresponding to a unit change in the appropriate explanatory variable, conditional on the other variables remaining constant. The intercept corresponds to the expected value of the response variable y when all the explanatory variables are zero. Significance tests of whether the coefficients take the value zero can be derived on the assumption that for a given set of values of the explanatory variables, y has a normal distribution with constant variance. The data was centered and scaled previous to applying logistic regression. Centering is performed by subtracting the column means (omitting missing values) of x from their corresponding columns, and scaling is done by dividing the (centered) columns of x by their root-mean-square. Explanatory variables were considered statistically significant when P < 0.05.

## Results

### Characterization of monoclonal YB-1-specific antibody F-E2G5

**Figure 1:**
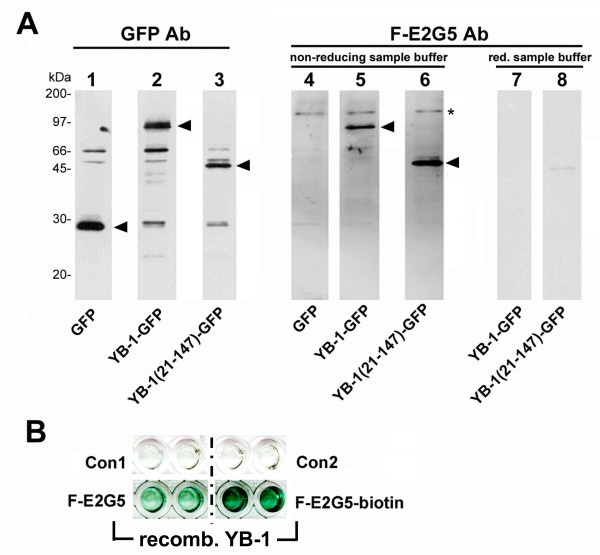
**Detection of YB-1-GFP and YB-1(21-147)-GFP by immunoblot and recombinant YB-1 by ELISA**. (A) HEK293T cell extracts containing GFP (lanes 1 and 4), YB-1-GFP (lane 2, 5 and 7) or YB-1(21-147)-GFP proteins (lanes 3, 6 and 8) were separated in denaturing gels and following transfer to nitrocellulose probed with GFP antibody (lanes 1 to 3) or Mab F-E2G5 (lanes 4 to 8). Relative mobilities of expressed proteins are indicated by arrowheads. Notably, reducing sample buffer prevents detection of tagged YB-1 proteins by immunoblot (lanes 7 and 8). (B) ELISA was performed by coating with recombinant affinity purified hexahistidin-tagged YB-1 protein. Biotin-labeled Mab F-E2G5 yields an even stronger signal than non-biotinylated Mab. Controls included omission of antibody (Con1) or antigen (Con2).

In a first approach the F-E2G5 antibody was examined for its substrate specificity by western blot analysis using cell extracts from HEK293 cells expressing GFP, YB-1-GFP or YB-1(21-147)-GFP proteins. GFP and YB-1-GFP fusion protein expression was ascertained by detection with monoclonal GFP antibody (Figure 1A, lanes 1-3). Immunoblotting with biotinylated F-E2G5 Mab was performed following addition of non-reducing (lanes 4-6) or reducing buffer (lanes 7-8) to protein samples. Mab F-E2G5 detects full-length YB-1-GFP fusion protein and also the truncated protein encompassing amino acids 21-147. However, detection succeeds only with non-reducing buffer and not under reducing buffer conditions with inclusion of mercaptoethanol (Figure 1A, compare lanes 5 and 7). Endogenous YB-1 protein with a relative molecular weight of 50 kDa was not detected by immunoblotting using F-E2G5 antibody, neither in denaturing nor in non-denaturing gels (data not shown). These results indicate that (i) the epitope(s) recognized by F-E2G5 resides within the YB-1 protein domains aa21-147 and (ii) detection is highly susceptible to the chosen conditions of western blotting.

One may conclude that the GFP-tag stabilizes the YB-1 protein conformation and permits successful detection by means of Mab F-E2G5, as the endogenous YB-1 protein from cell lysates (~50 kDa) was not detected by western blotting (only minute amounts were immunopositive following prolonged exposure). To ensure that Mab F-E2G5 successfully binds YB-1 protein under non-reducing and non-denaturing conditions, we thereafter established a direct ELISA with recombinant, affinity-purified YB-1 protein expressed in *E. coli*. Biotinylated as well as non-biotinylated Mabs F-E2G5 were tested. Under both conditions recombinant YB-1 protein was detected (Figure 1B), even better with biotinylated Mab F-E2G5. Controls included omission of antibody (Con1) or antigen (Con2) and addition of irrelevant IgG2/irrelevant biotinylated IgG2 antibody (not shown), all yielding negative results. Thus, Mab F-E2G5 binds epitope(s) that reside within YB-1 protein domains aa21 to 147. In addition, a sandwich ELISA was established with a series of GFP-tagged fusion proteins that include the following domains of YB-1 protein: aa21-147, aa146-317, aa146-225, aa146-172, aa260-317, aa146-262, aa21-262. Biotinylated Mab F-E2G5 was able to detect all fusion proteins that encompassed aa146-172. ELISA results were scored positive with an optical density of >0.4, whereas background values were below 0.05. These results support the notion that two distinct conformational epitopes are recognized by the Mab, one within the N-terminal (aa21-147) and one within the centrally localized domains (aa146-172).

**Figure 2:**
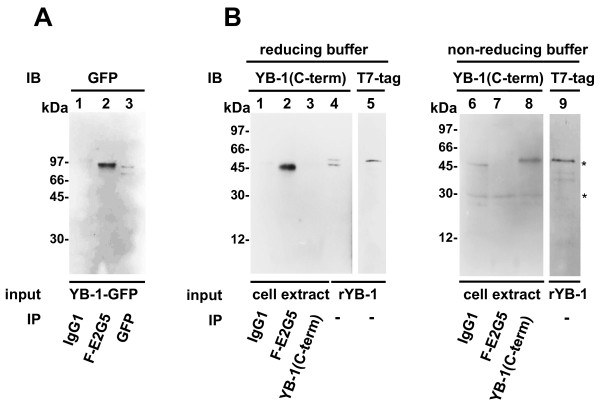
**Immunoprecipitation studies**. (A) Control IgG1, F-E2G5 and anti-GFP-tag antibodies were added to binding reactions with YB-1-GFP protein. Immunoblotting (IB) was carried out with anti-GFP antibody. (B) HEK293 cell protein extracts containing endogenous YB-1 protein were incubated with control IgG1, F-E2G5 and polyclonal anti-YB-1(C-term) antibodies and immunoprecipitated using pansorbin. Detection of protein pull-down was accomplished by immunoblotting with anti-YB-1(C-term) antibody. Sample buffers utilized were reducing (left) or non-reducing (right). Recombinant YB-1 protein was run aside w/o immunoprecipitation to visualize relative mobilities. "*" indicated mobilities of immunoglobulin heavy and light chains.

To further evaluate the propensity of Mab F-E2G5 to associate with YB-1-GFP or endogenous YB-1 proteins Mab F-E2G5 was evaluated in immunoprecipitation studies (Figures 2A and 2B). Mab F-E2G5 successfully pulled down YB-1-GFP, that was subsequently detected by monoclonal anti-GFP-tag antibody (Figure 2A). Furthermore Mab F-E2G5 immunoprecipitated endogenous YB-1 protein from HEK293 whole cell extracts, with immunoprecipitated protein being detected by polyclonal peptide-derived anti-YB-1 antibody directed against the protein C-terminus (denoted YB-1(C-term), Figure 2B, lane 2). Notably, when endogenous YB-1 protein was immunoprecipitated by polyclonal anti-YB-1(C-term) immunoblotting was unsuccessful under reducing conditions (Figure 2B, lane 3).

Next immunoprecipitated proteins were loaded on gels in non-reducing buffer (omission of mercaptoethanol and EDTA). Under these conditions immunoprecipitated endogenous YB-1 pulled down by monoclonal F-E2G5 antibody was no longer detected with polyclonal anti-YB-1(C-term) antibody. Successful immunoprecipiation by anti-YB-1(C-term) antibody was however possible (Figure 2B, lane 8). Heavy and light chains from immunoglobulins, that are contaminants from the immunoprecipitation procedure, were detected in samples loaded under non-reducing buffer conditions (indicated by "*" in Figure 2B).

From these results it is concluded that monoclonal F-E2G5 antibody has the propensity to immunoprecipitate YB-1-GFP as well as endogenous YB-1 protein, however, detection by means of immunoblotting with the utilized polyclonal anti-YB-1 antibody is susceptible to the chosen buffer conditions.

### Mab F-E2G5 is suitable for YB-1 immunodetection in formalin-fixed paraffin-embedded breast cancer tissue

**Figure 3:**
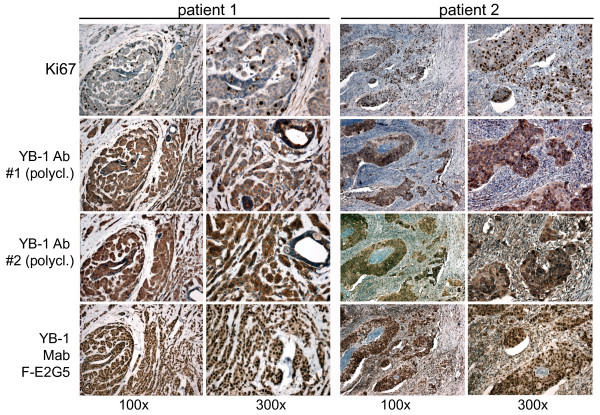
**Immunohistochemical comparison of different YB-1 antibodies in human invasive breast cancer**. While the proliferation marker Ki67 is strongly expressed in a defined fraction of breast tumor cells there is no concordance to the expression pattern of YB-1. The three YB-1 antibodies present very similar expression patterns. However, while the two established polyclonal YB-1 antibodies (#1 is against an N-terminal epitope and available from antibodies-online; #2 has been established by Janz et al. [1]) predominantly detect cytoplasmic YB-1 expression, the newly characterized monoclonal YB-1 antibody Mab F-E2G5 is able to detect nuclear YB-1 besides less abundant expression in the cytoplasm.

From our previous studies it was clear that the fixation procedure markedly affects detection of YB-1 in rat tissue [43]. In the following we performed immunohistochemistry of sequential tissue sections from patients diagnosed with invasive breast cancer. Biotinylated Mab F-E2G5 and two polyclonal peptide-derived YB-1 antibodies generated against distinct epitopes within the protein N-terminus were utilized. Notably, one of the polyclonal antibodies used (YB-1#2) has been previously applied by Janz et al. [1], the other is commercially available (YB-1#1; see Materials). Sequential sections were stained for Ki67 to identify cycling cells and exclude the possibility that biotinylated Mab F-E2G5 merely detects such cells. As can be seen in Figure 3 there was strong cytoplasmatic staining of cancerous cells with both polyclonal antibodies (#1 and #2). The overall pattern of immunopositive cells was similar with the Mab, however, a considerable fraction of cells exhibited strong nuclear staining. Ki67 positivity was observed in about 10 to 25% of all tumor cells, which was considerably lower than YB-1 positive cells detected by Mab F-E2G5. This finding excludes the possibility that the Mab only detects cycling cells.

From these results we conclude that the established Mab F-E2G5 is suitable for immunohistochemistry with formalin-fixed paraffin-embedded tissue, as routinely performed for histological analyses. The overall staining pattern with Mab and polyclonal antibodies is similar. However, Mab F-E2G5 preferentially detects nuclear YB-1.

### Nuclear YB-1 staining pattern in breast cancer tissue

**Figure 4:**
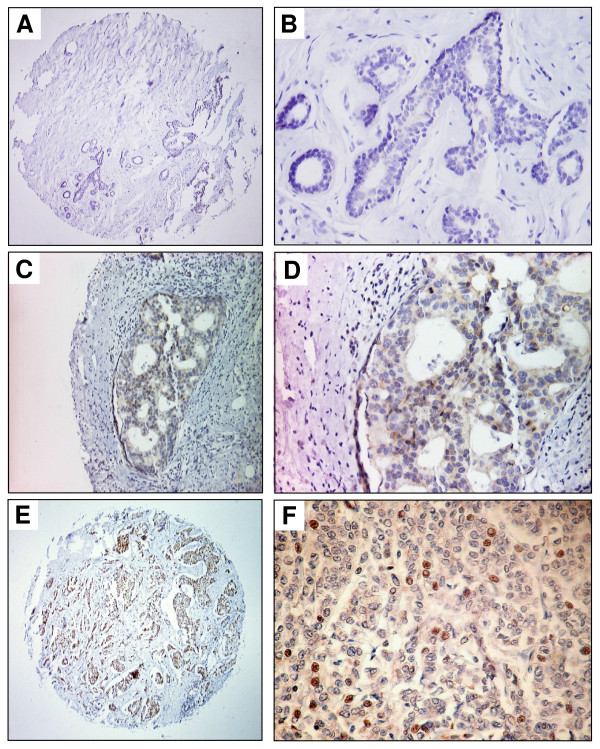
**Expression of YB-1 in normal breast tissue, non-invasive and invasive breast cancer**. (A) Only weak cytoplasmic YB-1 staining is detectable in normal breast tissue. (B) Scale-up of specimen shown in A. The magnification demonstrates weak YB-1 expression in luminal-epithelial breast cells. (C) Moderate YB-1 expression in a *ductal carcinoma in situ *(DCIS) of the breast. (D) Scale-up of specimen shown in C. (E) Invasive ductal breast cancer with abundant nuclear YB-1 expression. (F) Scale-up of specimen shown in E. Magnifications: A, C, E: 100×; B, D, F: 400×.

Immunohistochemical analysis was used to investigate YB-1 protein expression in normal breast tissue and breast cancers. We analyzed a tissue microarray with 224 breast tissue samples, i.e. 179 invasive ductal carcinomas, eight ductal carcinoma *in situ *(DCIS) and 37 normal breast tissue samples. To our knowledge, the latter group is larger than all other control groups analyzed by immunohistochemistry for YB-1 expression so far. Nuclear YB-1 immunohistochemical staining was only detectable in invasive ductal carcinomas, but not in DCIS and normal breast tissue. Figure 4 shows representative images of YB-1 expression in normal and malignant breast tissues. YB-1 staining was not detectable in normal breast tissue (Figures 4A and 4B). In DCIS (Figures 4C and 4D) cytoplasmic YB-1 expression was present in 75% (6/8) of cases, while nuclear YB-1 expression was not present. Invasive ductal carcinomas exhibited nuclear staining in 24% (43/179) of cases (Figures 4E and 4F).

### YB-1 expression, clinico-pathological parameters and patient survival

Clinicopathologic and immunohistochemical characteristics were correlated with nuclear and cytoplasmic YB-1 staining for descriptive data analysis (Table 1). Cytoplasmic YB-1 expression detected with the Mab F-E2G5 was not significantly correlated to overall (p = 0.134) and recurrence-free survival (p = 0.39).

**Table 1:** Clinico-pathological and immunohistochemical parameters in relation to YB-1 immunoreactivity, and univariate analysis of factors regarding tumor-related and recurrence-free surival

Variable	Categorization	Nuclear YB-1 immunoreactivity				Tumor-related death			Tumor recurrence		
		n analyzable	**neg**.	**pos**.	p*	n	events	p^†^	n	events	p^†^
***Clinico-pathological data:***											
Age at diagnosis											
	<50 years	51	43	8	**0.036**	51	9	**0.0064**	51	16	0.1895
	≥ 50 years	108	74	34		108	45		102	42	
Tumor stage											
	pT1	40	35	5	**0.004**	40	4	**<0.0001**	39	5	**0.0001**
	pT2	83	63	20		83	28		81	34	
	pT3	10	4	6		10	4		9	4	
	pT4	26	15	11		26	18		24	15	
Lymph node status											
	pN0	75	60	15	0.101	75	11	**<0.0001**	74	13	**<0.0001**
	pN1-3	80	54	26		80	41		76	42	
Histologic grade											
	G1	17	16	1	**0.011**	17	4	**0.0047**	17	4	**<0.0001**
	G2	69	55	14		69	17		65	16	
	G3	73	46	27		73	33		71	38	
Multifocality											
	unifocal tumor	141	104	37	1.000	141	45	0.0903	136	49	0.0683
	multifocal tumor	18	13	5		18	9		17	9	
Histologic type											
	ductal	125	93	32	0.710	125	43	0.9302	123	49	0.5730
	lobular	16	11	5		16	5		14	4	
	other	15	10	5		15	5		13	4	
											
***Immunohistochemistry (IHC):***											
Estrogen receptor status											
	negative	42	30	12	1.000	42	19	**0.0129**	42	24	**0.0026**
	positive	96	69	27		96	25		92	27	
Progesterone receptor status											
	negative	98	63	35	**0.002**	98	40	**0.0045**	93	44	**0.0019**
	positive	46	41	5		46	8		46	9	
Cytokeratin 5/6 IHC											
	negative (0)	118	85	33	0.540	118	34	**0.0100**	114	38	**0.0349**
	positive (1+-3+)	41	32	9		41	20		39	20	
TP53 IHC											
	<10%	85	62	23	1.000	85	28	0.3823	82	28	0.4422
	≥ 10%	48	31	17		48	18		46	19	
HER2 IHC											
	negative (0-1+)	108	83	25	1.000	108	28	**0.0081**	102	31	**0.0008**
	positive (2+-3+)	35	21	14		35	18		35	20	

* Fisher's exact test (two-sided), bold face representing significant data.

^† ^Log rank test (two-sided), bold face representing significant data.

**Figure 5:**
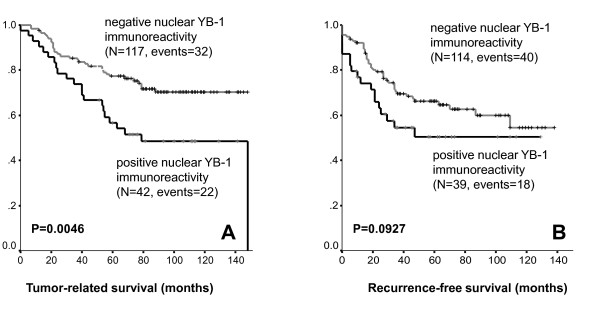
**A and B: Distribution of time (months) to tumor-related death (A) and tumor recurrence (B) among breast cancer patients with negative and positive YB-1 immunoreactivity as estimated by the method of Kaplan and Meier**.

**Figure 6:**
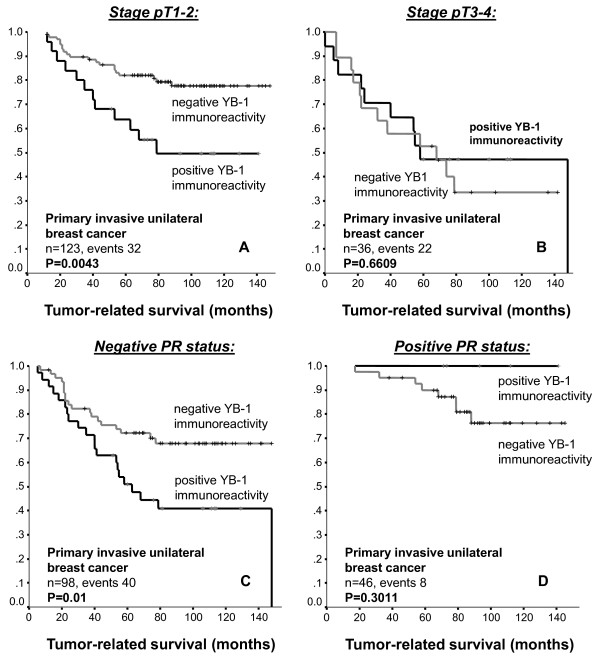
**A -- D: Distribution of time (months) to tumor-related death among the various subgroups (A, pT1-2; B, pT3-4; C, negative progesterone receptor status; D; positive progesterone receptor status) of breast cancer patients with negative and positive YB-1 immunoreactivity as estimated by the method of Kaplan and Meier**.

**Figure 7:**
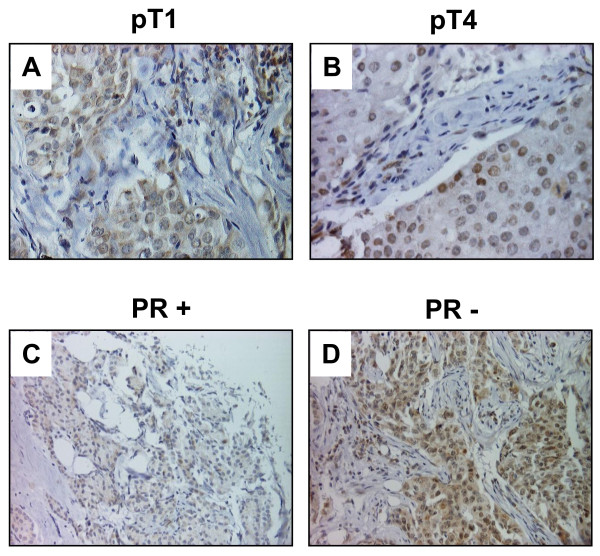
**Representative tissue microarray staining results illustrating YB-1 expression in relation to tumor stage (A and B) and progesterone receptor status (C and D)**. A and B: While YB-1 staining in small (pT1) tumors is predominantly cytoplasmic (A), large (pT4) tumors preferentially show a distinct condensation of YB-1 signal in the nuclei of tumor cells (B). C and D: In breast tumors with positive progesterone receptor status (C) YB-1 expression is predominantly cytoplasmic, while tumors that lost progesterone receptor expression (D) exhibit prominent nuclear YB-1 expression.

However, nuclear YB-1 expression detected by antibody F-E2G5 was associated with shorter overall survival (OS; p = 0.0046) and exhibited a strong trend towards association with shorter recurrence-free survival (RFS; p = 0.09). These were compared by Kaplan Meier analysis between invasive breast tumours with nuclear YB-1 expression *versus *all other invasive breast tumours exhibiting no nuclear expression (Figure 5). Patients with nuclear YB-1 expression in the tumor had an estimated mean OS of 90 months (95% confidence interval (CI): 72-109 months) compared to 117 months (95% CI: 108-126 months) in patients with absent nuclear YB-1 immunoreactivity. Nuclear YB-1 detection also correlated with tumor stage (p = 0.004), higher (G2/G3) histological grade (p = 0.011) and negativity of progesterone receptor status (p = 0.002) (Table 1). These strong correlations are demonstrated in Figure 6. In a stratified univariate analysis, the prognostic value of nuclear YB-1 detection became even more pronounced in the clinically important subgroup of stage pT1/T2 tumors, representing ~80% of all diagnosed carcinomas, and breast tumors with negative progesterone receptor status (Figures 6 and 7). While pT1 tumours exhibit no prominent nuclear YB-1 staining (Figure 7A), distinct nuclear YB-1 staining was detectable in pT4 tumours (B). In a similar comparison nuclear YB-1 protein was not detectable in most progesterone receptor positive breast tumours (Figure 7C), while there was prominent nuclear YB-1 staining in most tumors with negative progesterone receptor status (D).

Cox regression models including factors possibly influencing tumor-related survival and recurrence-free survival in relation to nuclear YB-1 expression were applied. As could be expected due to the size of our cohort, this analysis failed to demonstrate significance for nuclear YB1 detection as an independent prognostic marker (Table 2). Subsequently we analyzed nuclear YB-1 expression with respect to the molecular breast cancer subtypes defined by Perou and Sorlie [44,45]. A multiple logistic regression model was calculated with nuclear YB-1 expression as target variable and CK5/6, ER and HER2 as covariates (Table 3). None of the tested covariates remained significant; i.e., none of the covariates was significantly correlated with nuclear YB-1 expression, arguing that YB-1 is not selectively expressed in a specific molecular subtype of breast cancer.

**Table 2:** Multivariate Cox regression analysis of factors possibly influencing tumor-related survival and recurrence-free survival

				**Tumor-related survival**				**Recurrence-free survival**			
**Name**	**Variables**		**Categorisation**	**Global**	**Stepwise reverse selection^†^**			**Global**	**Stepwise reverse selection^†^**		
				p*	**Hazard ratio**	**95% Confidence interval**	**p**	p*	**Hazard ratio**	**95% Confidence interval**	**p**
				
***Model with dichotomous covariables***											
T	Tumor stage	0	pT1-2	0.402		-		0.153		-	
		1	pT3-4								
N	Lymp node status	0	pN0	**0.002**	5.050	2.264-11.267	**<0.001**	**0.038**	2.934	1.303-6.607	**0.009**
		1	pN1-3								
G	Histologic grade	0	G1-2	0.136	1.875	0.946-3.715	0.072	**0.007**	3.188	1.442-7.052	**0.004**
		1	G3								
ER	Estrogen receptor status	0	negative	0.168	0.420	0.213-0.829	**0.012**	**0.047**	0.357	0.173-0.733	**0.005**
		1	positive								
HER2	HER2 IHC	0	negative (0-1+)	0.386		-		**0.045**	2.164	1.019-4.594	**0.045**
		1	positive (2+-3+)								
CK5/6	Cytokeratin 5/6 IHC	0	negative (0)	**0.085**		-		0.305		-	
		1	positive (1+-3+)								
TP53	TP53 IHC	0	<10%	0.607		-		**0.043**	0.529	0.256-1.092	0.085
		1	≥ 10%								
YB1	YB1 IHC	0	negative (0)	0.530		-		0.738		-	
	1	positive (1+-3+)									

**Table 3:** Multiple logistic regression model.

	Estimate	Std. Error	t value	Pr(>| t|)
(Intercept)	-1.187	0.4558	-2.603	0.009
CK56	0.032	0.2460	0.131	0.896
ER	-0.009	0.0452	-0.207	0.836
HER2	0.226	0.1577	1.434	0.152

Null Deviance: 170.1; Residual Deviance: 167.8; AIC: 175.8; Degrees of Freedom: 146

The estimate for the regression coefficients show the change in YB-1 corresponding to a unit change in the corresponding marker (ER, HER2, CK5/6), conditional on the other markers remaining constant. No significant correlation can be seen between YB-1 and one of the other markers.

## Discussion

Major challenges in cancer diagnostics relate to early detection of malignant tissue and the possibility to predict the rate of disease progression and sensitivity to chemotherapy. Regarding all these issues the Y-box protein-1 may gain a prominent role, given that (i) it has an oncogenic property with induction of breast tumors in 100% of transgenic animals overexpressing YB-1 in the mammary gland [23], (ii) analyses of dysplasia-associated lesions in colitis ulcerosa patients reveal that lesions with increased cancer risk are distinguishable due to their YB-1 expression pattern [46], (iii) several reports on breast cancer indicate that YB-1 expression levels are strongly predictive for relapse rates and negatively correlate with disease free survival [6,34,36,47-50], and (iv) YB-1 upregulated P-glycoprotein expression. To date, however, no clear-cut standard for the assessment of YB-1 expression levels is available. In most immunohistochemical analyses overall expression levels have been scored in cancer tissues. Janz et al. even included the YB-1 expression within the adjacent tissue for analyses, which profoundly improved the predictive value on relapse-free survival [1].

One of the major obstacles towards a unifying scoring system in cancer tissue is the lack of a common expression pattern observed with diverse antibodies directed against YB-1. Most antibodies provide a highly specific staining pattern that is confined to the cytoplasm with complete absence within the nuclear compartment, as also reported in the most recent and largest study performed hitherto [6]. On the other hand, most - if not even all - activities of YB-1, that relate to oncogenic transformation, cell proliferation and drug resistance, must take place within the nuclear compartment. These are accompanied by altered gene transcription as well as chromosomal instability [18,20,23]. Thus, it is conceivable that a sensitive detection system and valid predictive testing must include such information, which is also reiterated by the observation of nuclear YB-1 being associated with P-glycoprotein expression and drug resistance [8,11]. It becomes even more complex when one envisions that YB-1 is a quite abundant cytoplasmic protein and has been detected in the nucleus in non-transformed cells as well, when tissue is fixed in Carnoys fixative [43]. The question is therefore, how these inconsistencies may be reconciled. One answer may be that YB-1 in cancer tissue has a different antigenicity due to post-translational modifications, e.g. phosphorylation of serine 102 [27]. In line with this interpretation is the observation of nuclear as well as cytoplasmic YB-1 in most cells using immunoblotting and senescence of cells with YB-1 *knock-out *[18,51]. Alternatively, cleavage of YB-1 within the protein C-terminus, as proposed before via the 20S proteasome [52,53], may render epitopes of YB-1 accessible for antibodies and result in positive immunodetection. Sorokin et al. have nicely demonstrated that the cleaved N-terminal fragment itself may shuttle to the nucleus and predict chemoresistance, whereas the overall full-length YB-1 expression level was unchanged in chemotherapy resistant versus chemosensitive cells. Attempts to explain the hidden secret beyond the different detection patterns observed already hint at the difficulties that the design and establishment of a monoclonal antibody to detect YB-1 faces.

In this report, we describe the generation of a monoclonal YB-1 antibody, termed F-E2G5, which detects YB-1 in formaldehyde-fixed paraffin-embedded tissues. Specificity of YB-1 detection by this antibody was confirmed by immunoblotting with YB-1-GFP fusion protein and ELISA. One detected epitope must reside within the central domains encompassing amino acids 21-147, a second one within domains aa146-172. Notably, using this antibody endogenous YB-1 protein is not detected by immunoblotting, likely due to its "misfolding" under the given conditions. Likely, that the GFP-tag within the protein C-terminus stabilizes the recognized epitope(s) for detection by western blotting. A non-specific band detected under some immunoblotting conditions does not seem to be of relevance for the performed immunohistochemistry, given that tissue specimens from healthy breast tissue do not demonstrate immunopositivity with Mab F-E2G5 and correlation results indicate that the outcome correlates with a nuclear staining pattern.

We have used this antibody to analyze YB-1 expression in a large and clinically well characterized collection of breast cancer specimens [41] that has previously been successfully used to define novel breast tumor markers [40]. Unlike Abba et al. [54] who described an absence of YB-1 mRNA expression in DCIS compared to normal tissues, we found an increased cytoplasmic concentration of YB-1 protein in six of eight analyzed DCIS compared to normal tissue. In our study, nuclear YB-1 expression was restricted to invasive ductal carcinomas. Conversely, tissue sections representing DCIS or normal breast tissue did not show nuclear expression of YB-1. Concordantly with these observations, nuclear expression of YB-1 in invasive breast cancer was significantly associated with overall survival (p = 0.0046). In our cohort this correlation was more predictive as observed by Janz et al. [1], who found a less tight association between YB-1-linked tumor aggressiveness and poor clinical outcome (p = 0.011).

So far we are the first group that describes a correlation between nuclear YB-1 expression and increased tumor grading (p = 0.011) and tumor stage (p = 0.004) in breast cancer. Although this does not necessarily reflect a causal relationship, it emphasizes the role of YB-1 as predictive marker. In non-small lung cancer and ovarian cancer and by applying polyclonal antibodies, other groups described an apparent link between YB-1 positivity in the nucleus and tumor staging. However, they did not find any correlation between YB-1 positivity and tumor grade in these entities [3,55,56].

A further finding of our study is the highly significant correlation between nuclear YB-1 expression and negativity of the progesterone receptor status (p = 0.002), which has not been described so far. Since YB-1 has been directly linked to intrinsic or acquired resistance to chemotherapy and thus might be responsible for failure of current treatment regimens, these findings implicate an interesting opportunity for the selection of chemotherapy resistant breast cancer patients out of the whole cohort of patients. ER-positive/PR-negative breast cancers are known to respond less well to selective ER modulator (SERM) therapy than ER-positive/PR-positive tumors [57]. Therefore YB-1 expressing breast cancer may have a general tendency to be hormone refractory, their relative neoplastic potential thus requiring chemotherapy, even if the tumors are of small size, low grade or estrogen receptor positive.

Our data are not in line with two reports, where YB-1 is more commonly found in ER negative breast tumors [6,26]. One may explain this with differences in antibody detection sensitivity, altered epitopes of YB-1 in the nuclear compartment, with the latter being the more likely reason.

In our stratified univariate analysis the prognostic value of nuclear YB-1 expression became even more pronounced in the clinically important subgroup of stage pT1/T2 tumors and breast tumors with negative progesterone receptor status, respectively. Routine mammography screening for breast cancer in women aged 51-70 is becoming a standard in the European Community. Thus, the number and frequency of small sized stage pT1/T2 breast tumors detected is steadily increasing. A YB-1 immunohistochemistry-based diagnostic test could help to identify young patients with small tumors but significant risk of relapse. We are confident that this novel monoclonal YB-1 antibody is of great value in performing prospective studies to establish YB-1 as a tumor marker in the management of human breast cancer. For this purpose large confirmative meta-analyses on independent breast cancer cohorts will be necessary. Therefore we would like to encourage other academic groups to validate our data on their own tumor collections.

## Conclusion

The newly established monoclonal anti-YB-1 antibody (clone F-E2G5) is suited to detect nuclear YB-1 expression and may be of great value for prospective studies to validate YB-1 as a prognostic marker and to adjust treatment in breast cancer patients.

## Abbreviations

AKT: protein kinase B; EGF: epidermal growth factor; ELISA: enzyme-linked immunosorbant assay; ER: estrogen receptor; GFP: green fluorescent protein; Mab: monoclonal antibody; PR: progesterone receptor; YB-1: Y-box binding protein-1.

## Competing interests

PRM is co-founder of antibodies-online and filed a patent relating to monoclonal anti-YB-1 antibody clone F-E2G5. The other authors declare that there are no competing financial or non-financial interests.

## Authors' contributions

ED and PRM designed the study, performed data analyses and drafted the manuscript, AE and RK carried out monoclonal antibody generation and immunoassays, FW and EB were involved in scoring of the tissue array and data interpretation, TF performed statistical analyses and was involved in drafting of the manuscript, PJW and AH were involved in tissue array collection, design and data interpretation, SD was involved in data interpretation and drafting of the manuscript, UK participated in the design of the study. PRM originally conceived the study. All authors read and approved the final manuscript.

## Pre-publication history

The pre-publication history for this paper can be accessed here:

http://www.biomedcentral.com/1471-2407/9/410/prepub
